# Impact of Maternal Age on Singleton Birthweight in Frozen Embryo Transfer Cycles

**DOI:** 10.3389/fendo.2022.830414

**Published:** 2022-03-08

**Authors:** Zhe-xin Ni, Kun-ming Wan, Zhi-hao Zhou, Yan-ping Kuang, Chao-qin Yu

**Affiliations:** ^1^ Department of Traditional Chinese Medicine Gynecology, Changhai Hospital, Naval Medical University, Shanghai, China; ^2^ Department of Assisted Reproduction, Shanghai Ninth People’s Hospital, Shanghai JiaoTong University School of Medicine, Shanghai, China

**Keywords:** assisted reproductive technology, frozen–thawed embryo transfer, vitrification, maternal age, birthweight

## Abstract

**Background:**

Previous studies have investigated the effect of maternal age on assisted reproductive technology success rates. However, little is known about the relationship between maternal age and neonatal birthweight in frozen embryo transfer (FET) cycles. Whether maternal age influences singleton birthweight in FET cycles remains to be elucidated.

**Methods:**

This study was conducted at a tertiary care center, involving singleton live births born to women undergoing frozen–thawed embryo transfer during the period from January 2010 to December 2017. A total of 12,565 women who fulfilled the inclusion criteria were enrolled and grouped into four groups according to the maternal age: <30, 30–34, 35–39, and ≥40 years old. A multivariable linear regression analysis was conducted to reveal the relationship between maternal age and neonatal birthweight with controlling for a number of potential confounders.

**Results:**

The highest proportions of low birthweight (LBW, 4.1%), high birthweight (1.2%), preterm birth (PTB, 5.9%), and very PTB (0.9%) were found in the group over 40 years old, but no significant difference was observed among the four groups. Additionally, the 35–39-year-old group had the highest rate of very LBW (0.6%), whereas the 30–34-year-old group had the lowest rate of small for gestational age (SGA, 2.7%). However, multivariate analyses revealed that neonatal outcomes including PTB, LBW, and SGA were similar between the different maternal age groups.

**Conclusion:**

Grouping with different maternal age was not associated with mean birthweight and Z-scores of singletons resulting from FET.

## Background

In the past decades, many women delay childbearing until after the age of 40. The decline of fertility of elderly women forced them to seek the help of assisted reproductive technology (ART). According to statistics by the European IVF-monitoring Consortium, the proportion of women over 40 years of age that became pregnant through *in vitro* fertilization or intracytoplasmic sperm injection (IVF/ICSI) in Europe is increasing annually. For example, in the UK, the proportion increased from 12.7% to 26% in 1997 to 2015, and the same trend was observed in other high-income countries (1, 2).

Having a healthy baby is the ultimate goal of ART treatment. Increased risk of adverse neonatal outcomes is believed to appear in IVF/ICSI-conceived babies, even singletons, such as preterm birth (PTB), low birthweight (LBW), and small for gestational age (SGA), when compared with babies that are conceived spontaneously (3). Maternal age is a frequently used predictor of PTB, LBW, and perinatal mortality in women who have conceived naturally (4–7), and a number of studies have well revealed the negative correlation between maternal age and neonatal outcomes (8, 9). Women who have a baby through ART are, on average, older than those who conceive spontaneously as these techniques are often applied in response to age-related infertility problems (10). However, little is known regarding the influence of maternal age on birthweight in vitrified–thawed embryo transfer cycles.

To our knowledge, only few studies have examined the effect of maternal age on birthweight with ART, included different kinds of treatments, and considered sufficient confounders (11–14). Furthermore, the published data, except for Lin’s study (14), exclusively focused on fresh IVF cycles, without ruling out the possibility of adverse fetal growth caused by a hypoestrogenic milieu. Of note, supraphysiological estrogen levels during ovarian stimulation can create a suboptimal peri-implantation environment for implantation and placentation, thus causing abnormal fetal growth including LBW and SGA (15, 16). Unlike fresh ovarian stimulation cycles, frozen embryo transfer (FET) seems to provide a more physiological uterine environment for early fetal development (17). Thus, the current study aims to explore the effect of maternal age on the birthweight of newborns conceived through embryo transfers during FET cycles.

## Methods

### Study Design and Population

This retrospective study involved women who had undergone FET during the period from January 2010 to December 2017, which was performed at the Department of Assisted Reproduction of the Ninth People’s Hospital of Shanghai Jiao Tong University School of Medicine. Women who met the following inclusion criteria were included in the study: BMI <30 kg/m^2^ and transfer of embryos resulting in a live singleton birth. In all FET cycles, no more than two embryos can be transferred. Furthermore, only the first live birth IVF/ICSI cycles were retained for women who had more than one delivery during the study period. The exclusion criteria were as follows: vanishing twin syndrome, congenital uterine malformations, and presence of submucosal fibroids or polyps and intramural fibroids >4 cm determined by ultrasound or hysteroscopy. Women with gestational diabetes, pregnancy-induced hypertension, and preeclampsia were excluded as these pregnancy-related factors may have a negative effect on intrauterine fetal growth. This study was approved by the institutional review board of the hospital and was conducted in accordance with the Helsinki Declaration. Informed consent was not required due to the retrospective nature of the study, and patients’ data were used anonymously.

### Laboratory Protocols

The procedures of ovarian stimulation, oocyte retrieval, and IVF/ICSI have been described in previous studies (18, 19). In brief, IVF or ICSI was conventionally performed according to semen parameters and previous fertilization histories. For IVF, oocytes were inseminated in human tubal fluid (HTF, Irvine Scientific), which was supplemented with 10% serum substitute supplement (SSS, Irvine Scientific) and ~300,000 progressively motile spermatozoa. For ICSI, oocytes were transferred into dishes immediately after microinjection with HTF+10% SSS. The assessment of fertilization was performed 16–18 h after insemination/injection. A dish containing pre-equilibrated culture medium was then prepared for the transfer of zygotes. Before 2013, embryos were cultured in Early Cleavage Medium (Irvine Scientific) before day 3 and then in MultiBlast Medium (Irvine Scientific). However, a continuous single culture medium (Irvine Scientific) was introduced after January 2013. All embryos were cultured under mineral oil and grown in an incubator at 37°C under 5% O_2_ and 6% CO_2_ concentration (the balance gas was nitrogen). Except for the change of culture medium types, no change was made for other laboratory conditions and IVF protocols throughout the study period.

### Endometrial Preparation and Vitrification

The endometrial preparation protocols for FET have been previously described (20). Briefly, a natural cycle FET was suitable for women having regular menstrual cycles with the use of HCG for triggering ovulation. Artificial cycles were offered for women with irregular cycles according to the discretion of treating physicians. The procedure of vitrification and thawing have been previously described (19). In short, the Cryotop carrier system with dimethyl sulfoxide–ethylene glycol–sucrose was used as cryoprotectants for embryo vitrification. Dilution solution in a sequential manner (1 mol/L to 0.5 mol/L to 0 mol/L sucrose) was used for thawing embryos. All embryos were thawed on the day of transfer.

### Maternal Age

Maternal age at the birth of the child was the key explanatory variable, which was divided into the following categories: <30, 30–34, 35–39, and ≥40 years old. The age group <30 years old was set as the reference category in our analyses.

### Outcome Measures

The primary neonatal outcomes focused on live singleton birthweight, including Z-scores, birthweight categories, and birthweight percentiles. Secondary outcome measures were associated with neonatal outcomes, including GA at birth and newborn gender. The definition for live singleton birth was a delivery of a singleton viable infant after the 24th gestational week. GA in FET cycles was calculated from the day of embryo transfer (day 17 for cleavage-stage embryo transfer and day 19 for blastocyst embryo transfer) (21). The definitions for PTB and very PTB were live births at <37 and <32 completed gestational weeks, respectively. Z-scores were calculated according to GA and newborn gender on birthweight based on the national birthweight reference as previously described (22, 23). A birth weight <2500 g was defined as LBW, and that <1500 g was defined as very LBW. The birthweight of infant was divided as follows: LBW (<2500 g), very LBW (<1500 g), high birthweight (HBW, >4500 g), and normal birthweight. Birthweight percentiles were also based on the national birthweight reference (23) and were divided into the following categories: SGA defined as birthweight <10th percentile, very SGA defined as birthweight <3rd percentile, large for gestational age (LGA) defined as birthweight >90th percentile, and very LGA defined as birthweight >97th percentile.

### Statistical Analysis

One-way analysis of variance was performed for continuous data, whereas Pearson’s chi-squared test or Fisher’s exact test was applied for categorical data. A *post hoc* Bonferroni correction was performed for multiple comparisons. The association between maternal age and neonatal outcomes was detected by multivariable logistic regression analysis, and the independent effect of maternal age on neonatal outcomes was analyzed by multiple linear regression.

The multivariable analyses included the following confounders: maternal BMI, paternal age, parity, infertility cause and duration, insemination method, type of endometrial preparation, endometrial thickness, year of treatment, and newborn gender. The continuous covariates (maternal BMI, paternal age, infertility duration, endometrial thickness, and year of treatment) and categorical covariates in multivariable models are listed in **Table 1**. Maternal age <30 years old was taken as a reference group in multivariable analyses. For the development of IVF techniques over time (24), a sensitivity analysis was performed on treating the year of treatment as a categorical variable. All analyses were conducted with SPSS Statistics (version 21.0), and *P* < 0.05 was considered to be statistically significant.

**Table 1:** Patient treatment and demographic characteristics according to maternal age.

	<30 yn=3586	30-34 yn=5461	35-39 yn=2861	≥40 yn=657	P value^a^	P value^b^	P value^c^
**Maternal Age (years)**	27.29 ± 1.63	31.93 ± 1.40	36.47 ± 1.34	41.31 ± 1.53	<0.001	<0.001	<0.001
**Maternal BMI (kg/m2)**					<0.001	<0.001	<0.001
**<18.5**	534 (14.9)	615 (11.3)	270 (9.4)	37 (5.6)			
**18.5-22.9**	2218 (61.9)	3448 (63.1)	1791 (62.6)	414 (63.0)			
**23-27.4**	729 (20.3)	1254 (23.0)	719 (25.1)	194 (29.5)			
**≥27.5**	105 (2.9)	144 (2.6)	81 (2.8)	12 (1.8)			
**Paternal Age (years)**	29.69 ± 3.53	33.74 ± 3.68	38.03 ± 4.29	42.62 ± 5.15	<0.001	<0.001	<0.001
**Infertility cause**					<0.001	<0.001	<0.001
Tubal factors	2126 (59.3)	3226 (59.1)	1788 (62.5)	321 (48.9)			
PCOS	391 (10.9)	425 (7.8)	97 (3.4)	12 (1.8)			
Endometriosis	255 (7.1)	466 (8.5)	280 (9.8)	57 (8.7)			
Diminished ovarian reserve.	61 (1.7)	121 (2.2)	117 (4.1)	142 (21.6)			
Uterine factors	52 (1.5)	112 (2.1)	65 (2.3)	17 (2.6)			
Male	513 (14.3)	675 (12.4)	294 (10.3)	67 (10.2)			
Unexplained	188 (5.2)	436 (8.0)	220 (7.7)	41 (6.2)			
**Parity**					<0.001	<0.001	<0.001
0	3489 (97.3)	5159 (94.5)	2478 (86.6)	471 (71.7)			
>0	97 (2.7)	302 (5.5)	383 (13.4)	186 (28.3)			
**Infertility duration (years)**	2.53 ± 1.82	3.29 ± 2.42	4.11 ± 3.41	4.70 ± 4.55	0.058	0.067	0.11
**FET cycle rank**					<0.001	<0.001	<0.001
First	2332 (65.0)	3112 (57.0)	1444 (50.5)	298 (45.4)			
High order	1254 (35.0)	2349 (43.0)	1417 (49.5)	359 (54.6)			
**Fertilization method**					0.118	<0.001	<0.001
IVF	2210 (61.6)	3425 (62.7)	1871 (65.4)	414 (63.0)			
ICSI	990 (27.6)	1407 (25.8)	762 (26.6)	225 (34.2)			
IVF+ICSI	386 (10.8)	629 (11.5)	228 (8.0)	18 (2.7)			
**Number of embryos transferred**					0.002	<0.001	<0.001
1	555 (15.5)	985 (18.0)	569 (19.9)	145 (22.1)			
≥2	3031 (84.5)	4476 (82.0)	2292 (80.1)	512 (77.9)			
**Embryo developmental stage at transfer**					0.815	0.607	0.200
Day 3	3010 (83.9)	4573 (83.7)	2415 (84.4)	565 (86.0)			
Day 5/6	576 (16.1)	888 (16.3)	446 (15.6)	92 (14.0)			
**FET endometrial preparation**					<0.001	<0.001	<0.001
Natural cycle	737 (20.6)	1338 (24.5)	750 (26.2)	176 (26.8)			
Artificial cycle	2849 (79.4)	4123 (75.5)	2111 (73.8)	481 (73.2)			
**Endometrial thickness (mm)**					0.040	<0.001	<0.001
<8	232 (6.5)	399 (7.3)	270 (9.4)	83 (12.6)			
8-11	1958 (54.6)	3067 (56.2)	1610 (56.3)	369 (56.2)			
>11	1396 (38.9)	1995 (36.5)	981 (34.3)	205 (31.2)			
**Year of treatment**					0.380	0.001	<0.001
2010–2012	276 (7.7)	465 (8.5)	218 (7.6)	33 (5.0)			
2013–2014	1693 (47.2)	2561 (46.9)	1219 (42.6)	250 (38.1)			
2015-2017	1617 (45.1)	2435 (44.6)	1424 (49.8)	375 (56.9)			

a 30-34 years old vs. <30 years old.

b 35-39 years old vs. <30 years old.

c ≥40 years old vs. <30 years old.

Data are presented as mean ± SD for continuous variables and n (%) for dichotomous variables.

## Results

The final dataset included 12,565 women who fulfilled the inclusion criteria, with no loss to follow-up. Baseline demographic and cycle characteristics are presented in **Table 1**. Comparison between the reference group and the other three groups revealed significant difference for maternal BMI, infertility cause, parity, infertility duration, FET cycle rank, fertilization method, number of embryo transferred, FET endometrial preparation, endometrial thickness, and year of treatment. Infertility duration and embryo developmental stage at transfer did not differ significantly among maternal age categories.

Neonatal outcomes stratified by maternal age are listed in **Table 2**. No significant difference was observed on GA and mean birthweight, and the gestation-adjusted Z-scores varied significantly according to maternal age categories. The 30–34-year-old group had the highest birthweight and Z-scores (3355.8 ± 483.3 g, 0.38 ± 1.03). With the increase of maternal age, a modest decrease of birthweight was observed, and the group over 40 years old had the lowest birthweight values (3321.6 ± 503.9 g). Additionally, no significant differences were found between any two groups by *post hoc* analysis on birthweight and Z-scores. The highest proportions of LBW (4.1%), HBW (1.2%), PTB (5.9%), and very PTB (0.9%) were found in the group over 40 years old, but no difference was observed among the four groups. Interestingly, the 35–39-year-old group had the highest rate of very LBW (0.6%), whereas the 30–34-year-old group had the lowest rate of SGA (2.7%). Furthermore, no difference was observed in very LGA, LGA, very SGA, and newborn gender between groups.

**Table 2:** Neonatal outcomes of live born singletons by maternal age.

	<30 yn=3586	30-34 yn=5461	35-39 yn=2861	≥40 yn=657	P value^a^	P value^b^	P value^c^
**Gestational age**					0.779	0.324	0.487
≥37 weeks	3367 (93.9)	5117 (93.7)	2694 (94.2)	612 (93.2)			
preterm birth (<37 weeks)	198 (5.5)	316 (5.8)	143 (5.0)	39 (5.9)			
very preterm birth (<32 weeks)	21 (0.6)	28 (0.5)	24 (0.8)	6 (0.9)			
**Birthweight (g)**	3352.2 ± 485.7	3355.8 ± 483.3	3334.2 ± 493.6	3321.6 ± 503.9	0.976	0.336	0.335
**Z-scores**	0.36 ± 1.04	0.38 ± 1.03	0.35 ± 1.06	0.38 ± 1.10	0.754	0.984	0.939
**Birthweight categories**					0.761	0.464	0.240
Very low birthweight (<1500 g)	12 (0.3)	19 (0.3)	17 (0.6)	2 (0.3)			
Low birthweight (<2500 g)	107 (3.0)	181 (3.3)	90 (3.1)	27 (4.1)			
High birthweight (>4500 g)	27 (0.8)	48 (0.9)	22 (0.8)	8 (1.2)			
**Birthweight percentiles**					0.186	0.498	0.937
Very small for gestational age (<3rd percentile)	48 (1.3)	83 (1.5)	35 (1.2)	9 (1.4)			
Small for gestational age (<10th percentile)	126 (3.5)	150 (2.7)	100 (3.5)	20 (3.0)			
Large for gestational age (>90th percentile)	369 (10.3)	583 (10.7)	326 (11.4)	74 (11.3)			
Very large of gestational age (>97th percentile)	258 (7.2)	354 (6.5)	183 (6.4)	46 (7.0)			
**Newborn gender**					0.667	0.564	0.393
Female	1881 (52.5)	2839 (52.0)	1522 (53.2)	349 (53.1)			
Male	1705 (47.5)	2622 (48.0)	1339 (46.8)	308 (46.9)			

a 30-34 years old vs. <30 years old.

b 35-39 years old vs. <30 years old.

c ≥40 years old vs. <30 years old.

Data are presented as mean ± SD for continuous variables and n (%) for dichotomous variables.

In multivariate analyses (**Table 3**), the neonatal outcomes including PTB, LBW, HBW, SGA, and LGA were similar between the different maternal age groups. The odds of PTB and LBW were lower in the group over 40 years old compared with the reference group, which did not reach a significant difference. Although the analysis of very PTB (<32 weeks) and very LBW (<1500 g) was performed, the number of cases in the two categories was too small to make any meaningful comparisons. In addition, no significant difference was found on birthweight percentile categories between the reference group and the other three groups.

**Table 3:** Odds ratios for gestational age and birth weights by maternal age.

	<30 yn=3586	30-34 yn=5461	35-39 yn=2861	≥40 yn=657
**Gestational age between < 37 weeks**				
Crude OR	Reference	0.952 (0.793-1.143)	1.108 (0.888-1.382)	0.923 (0.648-1.315)
Adjusted OR	Reference	0.951 (0.749-1.209)	1.096 (0.779-1.542)	0.947 (0.586-1.530)
**Gestational age < 32 weeks**				
Crude OR	Reference	1.140 (0.646-2.010)	0.700 (0.389-1.260)	0.636 (0.256-1.583)
Adjusted OR	Reference	1.015 (0.476-2.163)	0.825 (0.301-2.257)	0.741 (0.206-2.668)
**Very low birthweight (<1500 g)**				
Crude OR	Reference	0.957 (0.464-1.974)	0.561 (0.267-1.176)	1.081 (0.241-4.844)
Adjusted OR	Reference	1.132 (0.470-2.728)	0.701 (0.222-2.217)	1.211 (0.203-7.228)
**Low birthweight (<2500 g)**				
Crude OR	Reference	0.896 (0.703-1.142)	0.944 (0.710-1.255)	0.715 (0.465-1.100)
Adjusted OR	Reference	0.959 (0.703,1.309)	1.029 (0.662-1.599)	0.787 (0.435-1.423)
**High birthweight (>4500 g)**				
Crude OR	Reference	0.836 (0.521-1.339)	0.975 (0.554-1.716)	0.610 (0.276-1.350)
Adjusted OR	Reference	0.961 (0.537-1.716)	0.965 (0.413-2.256)	0.846 (0.256-2.791)
**Very small for gestational age (<3rd percentile)**				
Crude OR	Reference	0.891 (0.623-1.275)	1.092 (0.704-1.694)	0.973 (0.474-1.995)
Adjusted OR	Reference	0.890 (0.571-1.388)	0.824 (0.432-1.570)	0.653 (0.253-1.685)
**Small for gestational age (<10th percentile)**				
Crude OR	Reference	1.294 (1.016-1.648)	1.003 (0.767-1.312)	1.149 (0.710-1.859)
Adjusted OR	Reference	1.217 (0.877-1.260)	1.015 (0.655-1.573)	1.066 (0.564-2.017)
**Large for gestational age (>90th percentile)**				
Crude OR	Reference	0.975 (0.849-1.120)	0.901 (0.769-1.056)	0.910 (0.696-1.188)
Adjusted OR	Reference	1.051 (0.877-1.260)	1.026 (0.799-1.316)	1.034 (0.721-1.483)
**Very large of gestational age (>97th percentile)**				
Crude OR	Reference	1.123 (0.950-1.328)	1.122 (0.921-1.367)	1.023 (0.737-1.420)
Adjusted OR	Reference	1.071 (0.856-1.339)	1.052 (0.769-1.439)	0.983 (0.623-1.549)

Data are Odds Ratios (OR) with 95% confidence interval (CI). Analyses were adjusted for maternal BMI, paternal age, infertility cause, parity, infertility duration, FET cycle rank, infertility cause, fertilization method, number of embryos transferred, embryo developmental stage at transfer, the type of endometrial preparation, endometrial thickness, year of treatment, and newborn sex.

Multiple linear regression analyses were conducted to assess the relationship between maternal age and birthweight (**Table 4**). Even after correction for a number of potential confounders, no significant correlation was found between maternal age and neonatal birthweight. Moreover, maternal BMI (*P* < 0.001), parity (*P* < 0.001), FET cycle rank (*P* = 0.001), number of embryos transferred (*P* = 0.034), embryo developmental stage at transfer (*P* < 0.001), endometrial thickness (8–11 mm, *P* = 0.018; >11 mm, *P* = 0.001), year of treatment (*P* < 0.001), GA (*P* < 0.001), and newborn gender (*P* < 0.001) were all independent predictors for birthweight.

**Table 4:** Results of multiple regression analysis of singleton birthweight.

	Unstandardized coefficients	Std. error	Standardized coefficients	t	P value
	B		Beta		
**(Constant)**	-3532.855	99.754		-35.416	<0.001
**Maternal age**					
<30 (reference)					
30-34	-4.313	9.513	-0.004	-0.453	0.650
35-39	-14.879	13.020	-0.013	-1.143	0.253
≥40	-13.387	21.706	-0.006	-0.617	0.537
**Maternal BMI (kg/m^2^)**					
**<18.5**	-109.739	11.516	-0.072	-9.530	<0.001
**18.5-22.9 (reference)**					
**23-27.4**	86.312	8.825	0.075	9.781	<0.001
**≥27.5**	157.905	22.417	0.053	7.044	<0.001
**Paternal age**	1.556	0.932	0.017	1.669	0.095
**Parity, high order (versus 0)**	62.961	13.955	0.034	4.512	<0.001
**Infertility duration (years)**	1.282	1.364	0.007	0.940	0.347
**FET cycle rank, High order (versus First)**	26.116	7.516	0.027	3.474	0.001
**Infertility cause**					
Tubal factors (reference)					
PCOS	9.216	14.497	0.005	0.636	0.525
Endometriosis	2.194	13.273	0.001	0.165	0.869
Diminished ovarian reserve.	-10.895	20.651	-0.004	-0.528	0.598
Uterine factors	-10.179	26.186	-0.003	-0.389	0.697
Male	-14.409	12.591	-0.010	-1.144	0.252
Unexplained	-7.991	14.833	-0.004	-0.539	0.590
**Fertilization method**					
IVF (reference)					
ICSI	-18.255	9.320	-0.017	-1.959	0.050
VF+ICSI	4.260	12.769	0.003	0.334	0.739
**Number of embryos transferred, ≥2 (versus 1)**	22.384	10.540	0.018	2.124	0.034
**Embryo developmental stage at transfer, Day 5/6 (versus Day 3)**	71.030	11.139	0.053	6.377	<0.001
**FET endometrial preparation, Artificial cycle (versus Natural cycle)**	-15.143	8.558	-0.013	-1.769	0.077
**Endometrial thickness (mm)**					
<8 (reference)					
8-11	32.475	13.757	0.033	2.361	0.018
>11	45.989	14.243	0.045	3.229	0.001
**Year of treatment**					
2010–2012 (reference)					
2013–2015	-61.556	13.940	-0.063	-4.416	<0.001
2016-2017	-77.498	14.127	-0.079	-5.486	<0.001
**Gestational age (week)**	178.687	2.429	0.548	73.556	<0.001
**Newborn gender, female (versus male)**	-137.516	7.200	-0.141	-19.098	<0.001

## Discussion

In recent years, much attention has been paid to the impact of maternal age on ART success rates, and several studies have revealed that a high maternal age (over 40 years old) has a negative effect on pregnancy outcomes (11, 13). However, our study showed that no significant association existed between maternal age and singleton birthweight in FET cycles with consideration for related confounders. Furthermore, linear regression indicated that maternal age was not an independent predictor of singleton birthweight in FET cycles.

To our knowledge, four studies have analyzed the potential relationship between maternal age and neonatal birthweight. Wennberg *et al.* investigated the influence of maternal age on adverse maternal and neonatal outcomes following ART treatment and found that the risk of LBW and very LBW was significantly higher in ART than in spontaneous conception (SC) singletons in all ages up to maternal age of 40 years (LBW: aORs 1.44–2.35; VLBW: aORs 1.67–3.44). Additionally, when the analysis was restricted to maternal age >35 years, an increased risk of LBW existed for SC pregnancies, but not for ART pregnancies (11). Due to medical, educational, and socioeconomic reasons, women aged >35 years who conceive through ART may pay more attention to their state of health and seek medical assistance more often than SC women, which could result in increased detection of complications and decreased risk of LBW. Second, Moaddab *et al.* found that maternal age did not predict newborns’ birthweight in pregnancies with maternal age grouping as <40, 40–44, 45–49, and ≥50 years old (12). However, the analysis on birthweight in the maternal age group <40 years old per se is inadequate, and important information is missing. There may be some interesting findings among groups at ages <25, 26–30, 31–34, and 35–40 years old, and the increased risk of LBW may appear at the age of 35 years old. However, this study seemed to miss this potential information. Many studies have set up more detailed groups with maternal age under 40 years old to assess the influence of maternal age on neonatal outcomes and gained more credible results (13, 14). Another study reported that the risk of LBW was increased only at maternal ages over 40 years old (6 percentage points, 95% CI: 0.2, 12) with medically assisted reproduction (MAR) compared with the risk of LBW at ages 30–34 years old (13). However, a limited number of confounders were included in the study, and the effect of different kinds of MAR treatments could not be reliably distinguished, which included less invasive treatments such as ovulation induction only that were less strongly associated with adverse birth outcomes (25). A recent study based on 4958 infertile women using a freeze-all strategy observed that maternal age grouping was not related with increased risks of LBW, very LBW, preterm LBW, and macrosomia (14). However, only 1450 singleton live births were involved for the analysis of LBW, and the 44–50-year-old group of singleton live births was very small (n = 9), which may limit the power of statistical analyses between groups.

Our study aimed to improve on the flaws of the abovementioned studies and focused on the exact role of maternal age in singleton birthweight after FET cycles. The current study based on 12,565 singleton newborns born after FET cycles demonstrated that maternal age itself had no impact on singleton birthweight, and neonatal outcomes including PTB, LBW, and SGA were similar between the different maternal age groups. Due to several confounding factors, direct comparability across different age groups has very limited clinical significance in **Table 2**. Because of the strict exclusion criteria, the generality of this finding may be, to some extent, restricted. The reason why no significant correlation existed between maternal age and birthweight in our study is likely complex. It is generally known that with aging comes a reduction in ovarian function, resulting in the decrease of ovarian response to ovulation-promoting drugs and the low number of oocytes retrieved (26). Additionally, the decreased quality of oocytes (27, 28), abnormal endometrial function, and degeneration of multiple organ function will appear in women with advanced maternal age (22, 29). All the above-mentioned factors would affect the development of the embryo and cause adverse effects on the newborn, leading to LBW. However, with the popularization of education, many women tend to choose late marriage and late childbearing and enjoy a simple single life before marriage. In this kind of life, they are less stressed and have more opportunities to get in touch with life than women who are married and have children. Meanwhile, these knowledgeable women tend to choose a healthy and regular life and possess good habits, physical quality, and economic conditions, thus having a better choice on ART treatment (11). Aging leads to an irreversible decline in fertility, forcing older women to pay more attention to pregnancy and to seek medical help more actively than young women. In addition, the spouse’s income increases with age to guarantee maternity. Most importantly, the development of ART has well fulfilled the reproductive needs of women with different ages to improve the quality of newborns.

In this study, the results of multiple linear regression analysis indicated that maternal BMI, embryo developmental stage at transfer, parity, number of embryos transferred, endometrial thickness, year of treatment, GA, and newborn gender were independent predictors for neonatal birthweight, which was consistent with previous results (22, 30, 31). Z-scores were calculated and compared across the four groups to reduce bias caused by newborn gender and GA, and no significant difference on Z-scores was found among different maternal age groups. In addition, significant differences were observed between the maternal age groups in baseline and cycle characteristics including infertility duration, infertility cause, and fertilization method. However, these confounders had no impact on neonatal birthweight based on the linear regression model.

This study has several limitations. The biggest one is its retrospective design, so we strictly checked the database with strict criteria. Second, due to personal privacy restrictions, we were unable to obtain the education and economic background of patients. Third, many confounding factors were strongly associated with birthweight in linear regression analysis. Data bias during the experimental design cannot be all corrected by regression equations. Furthermore, embryo quality and paternal BMI were important factors that may affect neonatal outcomes, yet these data were missed in this study. However, the large number of singleton live births from a single center can assure the practice consistency, which is the main strength of the current study. Additionally, aside from the change of culture medium types, all other laboratory conditions and protocols remained invariant throughout the study period. Furthermore, maternal age was recorded according to the identification card, and endometrial thickness was measured by the same trained sonographers, reducing recorder variability. Importantly, a number of potential confounders were included in our study, which may minimize their impact on the findings.

## Conclusions

This study expands the current knowledge about the association between maternal age and neonatal outcomes and shows that maternal age is not associated with mean birthweight and Z-scores. This important finding should be adequately applied for women over 40 years old prior to FET and strengthen their confidence. A large prospective study, of course, is needed to verify our findings in the future.

## Data Availability Statement

The original contributions presented in the study are included in the article/supplementary material. Further inquiries can be directed to the corresponding authors.

## Ethics Statement

This study was approved by the institutional review board of the Ninth People's Hospital of Shanghai Jiao Tong University School of Medicine (reference number 2017-211) and the Changhai Hospital of Naval Medical University (reference number CHEC 2019-100), and was carried out in accordance with the Helsinki Declaration. Due to the retrospective nature, informed consent was not required, and patients' data were used anonymously.

## Author Contributions

C-qY and Y-pK conceived and designed this study. Z-xN, K-mW, and Z-hZ contributed to data acquisition, analysis and interpretation, and drafted the manuscript. K-mW and Z-xN were responsible for the collection of data. All authors interpreted the data. All authors contributed to the article and approved the submitted version.

## Funding

This work was supported by the National Natural Science Foundation of China (81774352, 81703874).

## Conflict of Interest

The authors declare that the research was conducted in the absence of any commercial or financial relationships that could be construed as a potential conflict of interest.

## Publisher’s Note

All claims expressed in this article are solely those of the authors and do not necessarily represent those of their affiliated organizations, or those of the publisher, the editors and the reviewers. Any product that may be evaluated in this article, or claim that may be made by its manufacturer, is not guaranteed or endorsed by the publisher.
